# Sulfation pathways in the maintenance of functional beta-cell mass and implications for diabetes

**DOI:** 10.1042/EBC20240034

**Published:** 2024-12-04

**Authors:** Jonathan Wolf Mueller, Patricia Thomas, Louise Torp Dalgaard, Gabriela da Silva Xavier

**Affiliations:** 1 Department of Metabolism and Systems Science, University of Birmingham, Birmingham, U.K.; 2 Department of Science and Environment, Roskilde University, Roskilde, Denmark

**Keywords:** beta-cells, diabetes, insulin, sulfate activation, sulfation pathways

## Abstract

Diabetes Type 1 and Type 2 are widely occurring diseases. In spite of a vast amount of biomedical literature about diabetic processes in general, links to certain biological processes are only becoming evident these days. One such area of biology is the sulfation of small molecules, such as steroid hormones or metabolites from the gastrointestinal tract, as well as larger biomolecules, such as proteins and proteoglycans. Thus, modulating the physicochemical propensities of the different sulfate acceptors, resulting in enhanced solubility, expedited circulatory transit, or enhanced macromolecular interaction. This review lists evidence for the involvement of sulfation pathways in the maintenance of functional pancreatic beta-cell mass and the implications for diabetes, grouped into various classes of sulfated biomolecule. Complex heparan sulfates might play a role in the development and maintenance of beta-cells. The sulfolipids sulfatide and sulfo-cholesterol might contribute to beta-cell health. In beta-cells, there are only very few proteins with confirmed sulfation on some tyrosine residues, with the IRS4 molecule being one of them. Sulfated steroid hormones, such as estradiol-sulfate and vitamin-D-sulfate, may facilitate downstream steroid signaling in beta-cells, following de-sulfation. Indoxyl sulfate is a metabolite from the intestine, that causes kidney damage, contributing to diabetic kidney disease. Finally, from a technological perspective, there is heparan sulfate, heparin, and chondroitin sulfate, that all might be involved in next-generation beta-cell transplantation. Sulfation pathways may play a role in pancreatic beta-cells through multiple mechanisms. A more coherent understanding of sulfation pathways in diabetes will facilitate discussion and guide future research.

## Introduction

Type 1 and type 2 diabetes are complex disease entities, causing the morbidity and mortality of millions of people on our planet. These forms of diabetes are linked to deficient insulin production by the endocrine pancreas, leading to subsequent deregulation of glucose metabolism, and also lipid and protein metabolic pathways, manifesting as pathologies that affect multiple organs (extensively covered in [1]). The deficiency in insulin in diabetes could be caused by the lack of insulin-producing pancreatic β-cells – either by autoimmune destruction of said cells in type 1 diabetes or defective development of such cells, or by a decline in functional pancreatic β-cell mass caused by cell stress followed by cell death (as in Type 2 diabetes), where loss of function manifests as aberrant production and/or release of the hormone insulin from the β-cells. Thus, strategies for treatment of diabetes have generally been focused on increasing the sensitivity of target tissues to insulin, on increasing the function of existing/remaining pancreatic β-cells, or on replacement of β-cells.

A survey of the literature on publicly available databases showed that aberrations in sulfation pathways [2] have been variously linked to liver diseases (e.g. hepatocellular carcinoma, liver fibrosis, drug induced liver injury) [3], skeletal dysplasia, chondrodysplasias, and growth disorders [4], colitis and colonic carcinogenesis [5], migraines, autism [6]. The link between sulfation pathways and diabetes is a relatively recent one. Sulfation pathways are about making and breaking esters of sulfuric acid and various biomolecules [7]. They are the non-reducing branch of overall sulfur metabolism [8]; until recently an underexploited area of life-science research [9]. In all living organisms, sulfation pathways need sulfate in an activated form, and 3′-phospho-adenosine-5′-phospho-sulfate (PAPS) is the near-universal sulfuryl donor for cells to accomplish this task [10,11].

There are more than 50 sulfotransferase genes encoded in the human genome [7]. They all bind the PAPS cofactor in a similar manner, but they employ strikingly different molecular ways to recognize and bind a large variety of substrates [12,13]. Sulfation targets can be low-molecular weight compounds, such as steroids, other hormones, metabolites, or drugs. Interestingly, also lipids can be sulfated – a prominent sulfo-lipid is the galacto-cerebroside sulfatide [14], where the modification with sulfate has become eponymous. In proteins, tyrosine side chains of peptides and proteins can be sulfated, post-translationally. Recently, sulfation has been added to the list of possible modifications of histone proteins [15]; and PAPS synthases are known to shuttle into the cellular nucleus [16]. What remains is the sulfation of complex proteoglycans and glycosaminoglycans; high-molecular-weight protein-bound sugar chains, found predominantly in the extracellular matrix, or attached to extracellular domains of transmembrane proteins. In humans, chondroitin-sulfate, heparan-sulfate and heparin, to some extent, belong to this category.

The biosynthesis of PAPS is energy-demanding, and, hence, upregulated expression of the actual enzymes for PAPS synthesis – PAPS synthases PAPSS1 and 2 – may indicate active sulfation processes, where PAPS-dependent sulfotransferases add sulfate moieties to various biomolecules. Publicly available bulk RNA-sequencing datasets [17,18] suggest that the gene for 3′-phosphoadenosine 5′-phosphosulfate synthase 2, *PAPSS2*, in humans, is notably more expressed in pancreatic islets compared with hepatocytes.

Type 2 diabetes mellitus is one of several established risk factors for the development of pancreatic ductal adenocarcinoma [19]. Carbohydrate sulfotransferase CHST15 was first reported as a predictive marker for pancreatic ductal adenocarcinoma [20], linked with worse prognosis [21]. Reciprocally, silencing of CHST15 gene expression blocks tumor growth in a T-cell-dependent manner in mice [22,23]. Other carbohydrate sulfotransferases might be linked to different malignancies [24]. These findings show that targeting sulfation pathways may improve prognosis for these tumors by inducing an immunological effect.

This review will link different aspects of diabetes and glucose tolerance to a number of sulfated biomolecules. Some intersectional aspects of this review are summarized in Table 1. There is scope for sulfation pathways to play a role in pancreatic β-cell function and therefore to be linked with the pathogenesis of diabetes or regulation of insulin secretion. We hope that a more coherent understanding of sulfation pathways in diabetes will facilitate discussion, enabling us to ask the right questions for future research.

**Table 1:** Cross-sectional involvement of sulfation pathways in pancreatic β-cells

Research finding	Reference (s)
**Pancreatic β-cell function and survival**	
Heparin and heparan sulfate proteoglycans are involved in pancreatic β-cell function and survival	[25,34,39]
Cholesterol sulfate protects β-cells against apoptosis under stressful conditions, thus preserving β-cell mass	[96]
Estrogens regulate β-cell survival, function, and proliferation	[98]
**Signaling**	
High-affinity binding to heparan sulfates differentiates paracrine signaling from systemic signaling, in the case of FGF signaling	[32,33]
α-cells and β-cells have specific heparan-sulfate sulfation patterns impacting paracrine signaling	[38]
Insulin receptor substrate 4 (IRS4) is tyrosine-sulfated on Tyr921 IRS4 plays a role in insulin receptor signaling, in addition to IRS1 and IRS2, and may be A putative substrate for IGFR1, with anti-apoptotic effects	[65,66,71]
**Insulin secretion**	
Heparan sulfate 3-O-sulfotransferase isoform-1 involved in insulin secretion	[37]
Sulfate-removing enzyme iduronate 2-sulfatase IDS linked to insulin content in mouse islets, and lysosomal degradation of secretory peptides	[46]
Murine Tpst2 over-expression and impaired insulin secretion were linked in the pancreatic islets of hypothyroid mice	[55]
Microtubules impact on the glucose-regulated release of insulin containing vesicles in pancreatic β-cells, mediated by vesicular transport and exocytosis	[72–76]
**β-cell development**	
Heparan sulfate synthesis involved in β-cell development and maintenance of β-cell mass, with impact on glucose tolerance and insulin secretory capacity	[40]
Sulfation during pancreas development balances differentiation and proliferation	[135]
Heparan sulfate supports postnatal mouse islet proliferation and function	[38,136]
Microtubules contribute to pancreatic β-cell heterogeneity	[77,78]
**β-cell** ** pathophysiology**	
Sulfated glycosaminoglycan, a substrate for the IDS enzyme, promotes the formation of amyloid from pro-islet amyloid polypeptide processing Intermediates *in vitro*, thus leading to loss of β-cell mass	[48]
Increased expression of vitamin-D-binding protein has been associated with dedifferentiation of β-cells and with β-cell dysfunction	[120,121]
Testosterone exposure during pregnancy caused defective β-cell programming in the offspring of various mammals	[107–111]
**Metabolism**	
Heparan sulfate glucosamine-6-O-endosulfatase-2 (Sulf2) in hepatocytes removes sulfate groups from syndecan-1-type heparan sulfate proteoglycans, thus reducing triglyceride-rich lipoprotein uptakes in cultured hepatocytes	[49]
Cholesterol sulfate regulates glucose metabolism, by inhibiting hepatic gluconeogenesis and lipogenesis, thus contributing to Type 2 diabetes	[94,95]
**Autoimmune diabetes**	
Heparan sulfates protect against autoimmune diabetes and immune cell infiltration	[34,51]
Heparan sulfates and other sulfated carbohydrates protect β-cells against an autoimmune response and help maintain a functional β-cell mass	[52]
Anti-inflammatory properties of the sulfatide C24:0 isoform protect β-cells from autoimmune attacks	[90]

## Heparan sulfates and islet function

Heparan sulfates are structurally extremely diverse carbohydrate chains, that are bound to an extracellular or transmembrane protein. The main classes of heparan sulfate are syndecans and the membrane-anchored glypicans. Heparin shares many similarities in the composition of carbohydrates and in the sulfation patterns; heparins however are not protein-bound. Heparin and heparan sulfate proteoglycans, as well as enzymes that degrade heparan sulfates, have been shown to be important in the regulation of pancreatic β-cell function and survival (reviewed in [25]). Heparan sulfate proteoglycans consist of heterogeneous, highly negatively charged polysaccharides, tethered to proteins on the cell surface or in the extracellular matrix [26]. The structural diversity of heparan sulfates is massive – rivaling that of proteins [27]. Heparan sulfate diversity is created from different heparan modifying enzymes acting in additive or mutually exclusive ways [12], such as the heparan sulfate sulfotransferases targeting the 2-OH, 3-OH, or 6-OH hydroxyl groups. Thus, patterns are created in heparan sulfate that are referred to as sulfation code [28,29].

Sulfated proteoglycans are important for cell development anywhere in the human body. These molecules act like inter-cellular lubricants; they also modulate how cytokines and growth factors distribute within tissue [30]. A striking example of what heparan sulfates can do to a signaling pathway are fibroblast growth factors (FGFs) [31]. More than 20 different FGF molecules target four different FGF receptors (FGFRs), but different binding affinities toward heparan sulfates make some of them endocrine or systemic signaling compounds [32]. The others cause paracrine signaling [33], spatially confined by heparan sulfates.

In the context of the Islets of Langerhans, heparan sulfate was found to be expressed at high levels in mouse pancreatic islets [34], with heparan sulfate proteoglycans concentrated in the intracellular space of mouse β-cells [35,36]. The sulfation pathway gene heparan sulfate 3-O-sulfotransferase isoform-1 has been shown to be involved in insulin secretion [37]. Differential sulfation patterns have been observed between α- and β-cells [38]. These specific heparan-sulfate-sulfation-patterns are thought to be important for paracrine signaling between islets cell types [38], potentially important for appropriate control of blood glucose levels.

Loss of heparan sulfate during culture of mouse islets was associated with increased β-cell death, which was reversed by culture with heparin (a highly sulfated, soluble variant of heparan sulfate) [34,39]. Disruption of heparan sulfate synthesis in mice led to reduced β-cell mass, and reduced insulin secretory capacity [40], potentially through an impact on β-cell development and maturation, culminating in impaired glucose tolerance. In humans, genetic variation in the EXT2 gene, which encodes for exostosin glycosyltransferase 2, an enzyme involved in the heparan sulfate biosynthesis, is associated with increased risk for Type 2 diabetes [41,42] and impaired glucose clearance [43]. Additionally, loss-of-function mutations in the EXT2 gene, and the related EXT1 gene, was shown to be associated with lowered insulin secretion and impaired β-cell reserve in humans, as assessed by hyper-glycemic clamp and arginine challenge [44]. Thus, these genetic data indicate that heparan sulfates are required for the development and maintenance of functional β-cell mass in humans.

Iduronate 2-sulfatase (IDS) is a lysosomal enzyme involved in the degradation of proteoglycans. Interrogation of the single-cell RNA-seq dataset generated by Camunas-Soler and colleagues [45] indicated that expression of the IDS gene is lowered by approximately 30% (pAdjusted, 1.62 × 10^−9^) in β-cells from people living with Type 2 diabetes versus β-cells from individuals not living with Type 2 diabetes. Reduction of murine Ids expression was previously associated with reduced insulin content in mouse islets, whilst the secretory response to glucose was maintained [46]. Due to its localization in the lysosomes in pancreatic islets, it was hypothesized that Ids may be involved in the lysosomal degradation of secretory peptides. *Ids* expression in mouse pancreatic islets is regulated by glucose [46] and over-expression of *Ids* in INS1E cells, a rat insulinoma cell line, enhanced glucose-stimulated insulin secretion via a pathway dependent on protein kinase C alpha [47]. Increased levels of sulfated glycosaminoglycan, a substrate for IDS, increase the formation of amyloid from pro-islet amyloid polypeptide processing intermediates *in vitro* [48]. Amyloid deposition in pancreatic β-cells leads to loss of β-cell mass.

Genetically diabetic (db/db) mice overexpress the enzyme, heparan sulfate glucosamine-6-O-endosulfatase-2 (Sulf2) in hepatocytes, which may remove sulfate groups from syndecan-1-type heparan sulfate proteoglycans [49]. This in turn leads to suppression of the uptake of atherogenic postprandial triglyceride-rich lipoproteins in cultured hepatocytes [49]. Inhibition of Sulf2 in db/db mice using antisense oligonucleotides was shown to normalize the capacity of hepatocytes to bind very-low density lipoprotein and abolish postprandial hypertriglyceridemia [50]. Sulf2 protein content in mouse liver and in cultured hepatocytes is suppressed by adiponectin and insulin [49]. Thus, appropriate sulfation of cell surface proteo-glycans is linked to mechanisms that regulate energy flux.

Finally, heparan sulfates may play a role in protection against autoimmune diabetes, as autoimmune destruction of islets of non-obese diabetic mice and in streptozotocin-induced diabetic mice was prevented by inhibition of heparinase – stopping heparan sulfates to be degraded thereby reduced immune cell infiltration [34,51]. Thus, the evidence points to the involvement of heparan sulfate in protection of β-cells against an autoimmune response and maintenance of a functional β-cell mass [52], indicating a potential role in the pathophysiology of both Type 1 and 2 diabetes.

## Sulfated proteins in diabetes

Tyrosine sulfation, a post-translational modification of proteins, is catalyzed by the tyrosyl-protein sulfotransferases TPST1 and TPST2 [7]. Tyrosine sulfation is a common modification found on secreted proteins as well as extracellular parts of transmembrane proteins [53]. In addition, intracellular protein sulfation was reported very recently for the very first time [15]. Tyrosine sulfation is important for protein-protein interactions and has been shown to be involved in the regulation of hemostasis, the inflammatory response and chemokine recognition [54].

TPST1 and 2 are expressed in pancreatic islets; in fact, both genes are among the highest-expressed sulfotransferases in β-cells, in publicly available datasets [17,18]. What is the evidence for a role for protein sulfation in the regulation of islet hormone production and action? Hypothyroid growth-retarded mice show impaired insulin secretion, and at the same time, murine Tpst2 is over-expressed in the pancreatic islets [55], suggesting a possible link between Tpst2 and insulin secretion. Indeed, increased sulfation was reported to contribute to atherosclerosis related to chronic kidney disease [56], a prevalent chronic complication in Type 2 diabetes.

**Figure 1:**
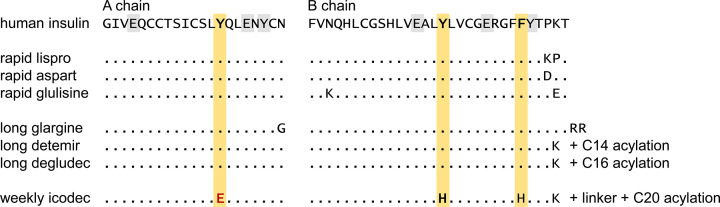
Short and long lasting insulin variants. With regard to their molecular composition, established rapid-acting and long-acting insulins are compared with the newly introduced once-weekly insulin icodec. TyrA14Glu, TyrB16His, and PheB25His are mutations not contained in any of the commonly used insulins so far. The C14 acylation of detemir is myristic acid; the C16 acylation in degludec is hexadecanedioic acid; only the C20 acylation in icodec is more complex, two oligoethylene glycol units connect LysB29 to an oligoethylene icosanedioic diacid, via a gamma-glutamate moiety. Please note that detemir, degludec, and icodec all are ΔThrB30 insulins. Rapid and long insulins are presented according to [145], the insulin icodec molecule is described in [64].

Could it be that insulin itself is sulfated? There are a total of four tyrosine residues in human pre-pro-insulin, all of them end up in the 51-amino-acids-comprising mature insulin, highlighted in Figure 1. PubMed lists the search phrase ‘sulfated insulin’ in 16 publications, all before 1998; reaching back to a study in 1947 [57]. In the past, human insulin was an extreme rarity; instead, insulin from the pancreases of various animals, mainly pork and beef, was available for use in humans. Synthetically sulfated insulin was a type of formulation, believed to reduce antigenic responses in diabetic patients with immune insulin resistance [58]. Although there were some promising reports around sulfated insulin [59,60], the use of sulfated insulin ceased toward the late 1990s [61], as recombinant human insulin became readily available.

Chemical sulfation of insulin mimics biological sulfation to a certain extent. Tyrosyl-protein sulfotransferase TPST1 and TPST2 enzymes modify proteins on selected tyrosine side chains [62], during the proteins transit through the Golgi network [53,63]. The TPST1 and TPST2 sulfating enzymes tend to recognize tyrosine residues surrounded by negatively charged residues, located in accessible and somewhat flexible parts of the protein, thus creating similar, but not overlapping tyrosine sulfation patterns.

Although no direct evidence for sulfation of native insulin can be found in the literature, a recently approved ultra-long-lasting version of insulin, weekly ‘icodec’, is noteworthy here. This insulin carries a long acyl chain at LysB29; additionally, icodec features three amino acid changes, TyrA14Glu, TyrB16His, and PheB25His, featured in Figure 1 [64]. These are unusual replacements, as they all affect aromatic residues. One in particular, TyrA14Glu, actually mimics the negatively charged side chain of tyrosine sulfate, enhancing solubility and slowing down pharmacokinetics.

**Figure 2:**
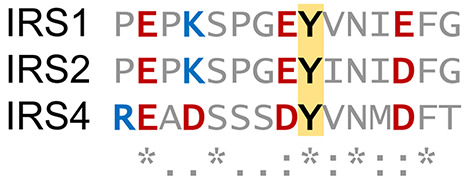
Alignment of different insulin receptor substrates. Protein sequences NP_005535 for IRS1, NP_003740 for IRS2, and NP_003595 for IRS4 were aligned using the multiple sequence alignment tool Muscle [146]. Sulfated IRS4 tyrosine Y921 corresponds to Y896 in IRS1 and Y919 in IRS2. Negatively charged amino acids depicted in red, positively charged ones, in blue.

Sulfation of other proteins may play a role in the regulation of β-cell function, supported by recent literature on mass-spectrometry surveys. Tyr921 of insulin receptor substrate 4 – IRS4 – was recently identified as a target for protein sulfation [65]; and IRS4 plays a role in insulin receptor signaling, in addition to IRS1 and IRS2. An alignment of this motif is shown in Figure 2. Alternatively, IRS4 is a putative substrate for IGFR1 [66]. Resistance to insulin and IGF1 in pancreatic β-cells has been shown to lead to compromised β-cell function [67–69]. Although the IRS1 and IRS2 are the major adaptor proteins involved in the transmission of the insulin signal (reviewed in [70]), over-expression of IRS4 has been shown to be protective against apoptosis in pancreatic β-cell lines lacking IRS2 [71].

Amongst the sulfated proteins identified by Daly and colleagues are also α- and β-tubulin, which are building blocks for microtubules. Microtubules are involved in vesicular transport and the regulation of exocytosis (reviewed in [72]); and this includes glucose-regulated release of insulin containing vesicles in pancreatic β-cells [73–76]. Microtubule also contribute to pancreatic β-cell heterogeneity [77,78].

Coming back to the link between protein sulfation and insulin secretion, the following sulfation pathway proteins have been identified in insulin-secretory-granules, in close proximity to pro-insulin and mature insulin [79]: Sulfated glycoprotein 1 precursor; arylsulfatase B; galactosamine (N-acetyl)-6-sulfate sulfatase; glucosamine (N-acetyl)-6-sulfatase. Arylsulfatase B and galactosamine (N-acetyl)-6-sulfate sulfatase are lysosomal enzymes involved in the breakdown of glycosaminoglycans. The expression of arylsulfatase B has been shown to be elevated in the liver of streptozotocin-induced diabetic rats [80]. This protein’s function, and likewise that of galactosamine (N-acetyl)-6-sulfate sulfatase, within the islet secretory vesicles is as yet unknown. However, they may, like the iduronate 2-sulfatase IDS, discussed above, be involved in the degradation of secretory peptidoglycan, thereby modulating β-cell function. Glucosamine (N-acetyl)-6-sulfatase is a lysosomal enzyme that is involved in the metabolism of heparin, heparan sulfate and keratan sulfate, and their involvement in the regulation of β-cell function and survival was discussed above. The sulfatide lipid is also present in exocytotic granules, as described further below.

Taken together, a number of sulfated proteins from within β-cells have been reported. With improved analytics, as recently shown [65], it is likely that more sulfated proteins will be reported from within islets.

## Sulfated lipids in insulin resistance and β-cell protection

Sulfation is important for the regulation of lipid metabolism and dysregulation of sulfation pathways have been shown to lead to dyslipidemia. Sulfated glycoprotein-1 is a saposin precursor (prosaposin); it is involved in the regulation of glycolipid metabolism, leading to ceramide production (reviewed in [81]. Ceramides are known to antagonize insulin action, thereby contributing to insulin resistance (reviewed in [82]). In the context of the pancreatic β-cell, elevated ceramide content is associated with increased β-cell apoptosis and defective insulin production and secretion (reviewed in [83]). Sulfation of ceramide heads is widespread in a special class of sulfated galacto-cerebroside, the sulfatides. Sulfatides are made by the cerebroside sulfotransferase GAL3ST1 and specifically degraded by arylsulfatase A [84]. Interestingly, arylsulfatase A was recently identified as regulator of sulfatide content and glycemic control [85], alongside the sphingolipid synthesizing enzyme hexosaminidase A [86].

Sulfatides can strongly influence the properties of the membranes they are part of [87]. Roeske-Nielsen and colleagues have identified a variation in the cerebroside sulfotransferase gene GAL3ST1 that is linked to exercise-modified insulin resistance and to Type 2 diabetes [88]. The same group also showed that sulfatide protects insulin-producing cells against cytokine-induced apoptosis [89]. A specific version of sulfatide, the C24:0 isoform, may have anti-inflammatory properties in the context of Type 1 diabetes, as it may protect β-cells from autoimmune attacks [90]. Finally, sulfatide might be a good addition to injectable commercial insulin formulations, because it strongly inhibits fibroblast growth [14], thus potentially reducing adverse fibroblast growth and improve well-being in patients with diabetes.

## Cholesterol sulfate and the membranes in the islet

Cholesterol is a central component of nearly all eukaryotic membranes. This sterol mainly gets sulfated by the cytoplasmic sulfotransferase SULT2B1b [91] and de-sulfated by the steroid sulfatase STS [7,92]. Rare genetic mutations in the human X-linked STS steroid sulfatase gene lead to a scaling of the skin, due to accumulation of cholesterol sulfate [5]. Cholesterol sulfate more and more is recognized as an emerging regulator of lipid metabolism, inflammation, and cell fate [93].

SULT2B1b and cholesterol sulfate regulate glucose metabolism, as cholesterol sulfate inhibits hepatic gluconeogenesis [94] and lipogenesis [95], thus contributing to Type 2 diabetes [94]. Mechanistic studies in rodents suggest, however, that cholesterol sulfate protects β-cells against apoptosis under stressful conditions, thus preserving β-cell mass and protecting against the development of diabetes mellitus [96]. The same authors reported cholesterol sulfate levels to be elevated in bodily samples from patients with Type 1 or Type 2 diabetes [96]. The knockout of the mouse Slc10a6 transporter surprisingly resulted in elevated serum levels of cholesterol sulfate [97]. Studying the role of sodium-dependent organic anion transporters (SOATs) might bring interesting new insights for cholesterol sulfate in the future. Because of downstream steroidogenesis, where cholesterol is converted to highly potent steroid hormones, causing their very own effects, studying cholesterol sulfate in molecular detail may be associated with unique challenges.

## Sulfated steroid hormones influence diabetes, and vice versa

The action of steroid hormones is regulated by their sulfation status [3]. There is evidence in the literature to indicate that sex steroids are involved in the regulation of pancreatic β-cell function and potential paracrine regulation (reviewed in [98]). The estrogen sulfotransferase SULT1E1 (also known as EST) catalyzes the sulfation of estrogens, temporarily inactivating these steroid hormones. SULT1E1 expression may be induced in inflammation-linked disease states, such as Type 2 diabetes, and some of its effects appear to be independent of estrogens [99], suggesting that other aromatic metabolites or steroids to be involved. The effects of the sulfation pathway gene SULT1E1 in energy homeostasis and insulin sensitivity may be sex- and tissue-specific [100,101].

Cells in the human islets of Langerhans express dedicated enzymes for the downstream conversion of steroid hormones [102,103], using sulfated steroid precursors from the circulation, such as DHEAS [3]. Estrogens in pancreatic β-cells have been shown to be involved in the regulation of β-cell survival, function, and proliferation in the context of both Type 1 and Type 2 diabetes, in rodent systems (reviewed in [98]). Activation of the androgen receptor [104] has been shown to augment insulin secretion in male [105], but to be deleterious to β-cell function in female rodent islets [106]. Offspring that had been exposed to high levels of testosterone during pregnancy showed defective β-cell programming in mice [107], sheep [108,109], and non-human primates [110,111]; findings that resemble what occurs in women with polycystic ovarian syndrome and their offsprings. It remains to be seen whether steroids currently under study, such as 11-oxo-androgens [5] or bis-sulfated steroid diols [112,113], play a role in the pancreatic β-cell as well.

## Vitamin D and diabetes

The literature linking vitamin D and diabetes is vast (reviewed in [114,115]), with evidence that vitamin D is important for the development [116], survival [117], and function [118,119] of pancreatic β-cells. We have previously shown [120] that loss of expression of vitamin D-binding protein, which carries vitamin-D-metabolites in the circulation, in mice, leads to elevated basal glucagon secretion but a decrease in glucagon secretion in response to glucose in pancreatic α-cells, which is paralleled by increased insulin secretion in response to glucose challenge, and that this is due to dysregulation of the actin-network [120]. In the islet, vitamin D-binding protein is primarily expressed in pancreatic α-cells [120], but its expression in pancreatic β-cells is induced by glucose and increased expression has been associated with dedifferentiation of β-cells and with β-cell dysfunction [121].

Sulfated vitamin D has only recently come into the focus of clinical analytics [122,123], with sulfated conjugates of vitamin D representing a high proportion of the circulating vitamin D metabolome. Several vitamin D metabolites are sulfated primarily by the cytoplasmic sulfotransferase SULT2A1 enzyme [124] in liver [125]. We now know that vitamin D-sulfates are a significant part of the vitamin D-metabolome both in serum [122,126] and breast milk [123]. However, it remains to be seen whether the sulfation status of vitamin D would impact β-cell function and/or glucose tolerance.

## Other sulfated molecules in diabetes – indoxyl sulfate as a metabolite from the intestine

The gut microbiota converts tryptophan from dietary protein to various metabolites. Indoxyl sulfate is one of the metabolites of tryptophan metabolism, produced by the intestinal bacterial flora and subsequent liver sulfation. Indoxyl sulfate has been mentioned many times in various metabolomics papers in recent years. Harlacher et al. linked indoxyl sulfate to an increase in inflammation, oxidative stress, leukocyte migration and adhesion, cell death and a thrombotic phenotype via the activation of the aryl-hydrocarbon-receptor [127]. Studies in human umbilical vein endothelial cells suggest that indoxyl sulfate may accelerate chronic kidney disease and drive vascular disease, by inducing oxidative stress and endothelial senescence [128]. In an animal model, indoxyl sulfate is taken up by proximal tubular cells through the organic anion transporters OAT1 and OAT3, and it induces reactive oxygen species (ROS) with impairment of cellular antioxidative system [128]. Via a number of signaling pathways, indoxyl sulfate induces nephro-vascular senescence [129].

**Figure 3:**
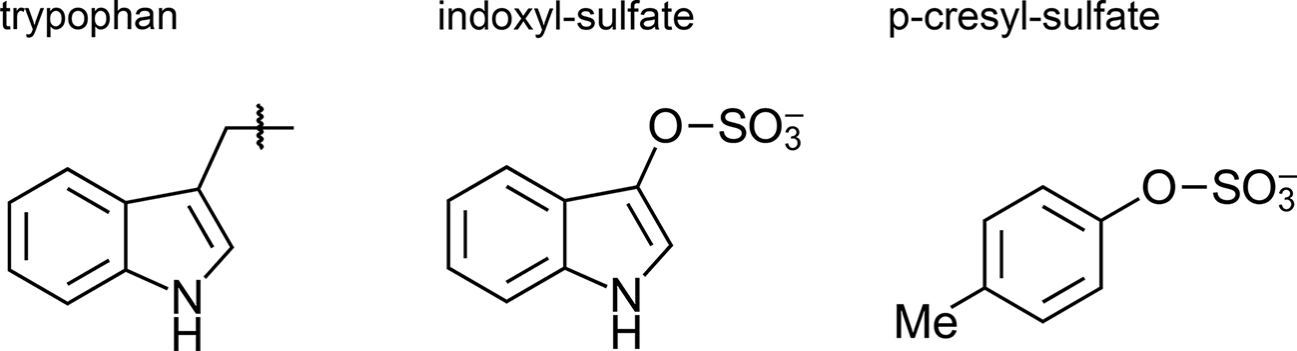
Indoxyl-sulfate and related metabolites. Molecular structures of tryptophan (showing the indole side chain only), of indoxyl-sulfate, and of para-cresyl-sulfate. Although indoxyl-sulfate and para-cresyl-sulfate are both produced in the intestine, they have different downstream effects.

In patients with Type 2 diabetes, indoxyl sulfate was linked to cardiovascular risk [130], and diabetic nephropathy [131]. In contrast with p-cresyl sulfate, indoxyl sulfate levels specifically correlate with glycated protein products in hemodialysis patients [132]; putatively increasing glucose toxicity and enhancing atherosclerosis (Figure 3). In patients with diabetes, urine indoxyl sulfate levels correlate with urinary markers of oxidative stress, suggesting a role as inducer of oxidative stress for indoxyl sulfate [133]. Studying sulfated metabolites, such as indoxyl sulfate, may reveal novel biomarkers to monitor disease progression. Furthermore, the elucidation of the signaling pathways around indoxyl sulfate toxicity may reveal new strategies for therapeutic intervention. Finally, even such simple biotechnological approaches as indole-absorbing matrices [134] for indoxyl sulfate removal might represent interesting avenues for future research and application.

## The biotech around pancreatic islets and cleverly sulfated polysaccharides

The potential of sulfation pathways might be harnessed in enabling technologies which could lead to better treatment for diabetes, particularly Type 1 diabetes. For example, understanding the regulation of sulfation during the development of pancreatic islets may be important in our efforts to generate pancreatic β-cells from embryonic stem cells or pluripotent stem cells for transplantation. Regulation of sulfation has been shown to be important during the development of the endocrine pancreas: inhibition of sulfation was reported to increase the balance toward differentiation versus proliferation in rodent embryonic pancreas [135]. Heparan sulfate has also been reported to be important for regulation of postnatal mouse islet proliferation and function [38,136]. Harnessing such pathways could be beneficial for the development of protocols that lead to efficient production of pancreatic β-cells in cell culture.

One of the big issues with islet transplantation is hypoxia-induced cell death in islets of Langerhans whilst in culture prior to transplantation. It has previously been shown that the culture of human and mouse islets in heparin leads to decreased islet cell death whilst in culture [34,137,138]. Indeed, heparin is administered during the transplant process for graft protection and to prevent blood coagulation at the graft site (UK Guidelines on Pancreas and Islet Transplantation 2019 https://bts.org.uk/wp-content/uploads/2019/09/FINAL-Pancreas-guidelines-FINAL-version-following-consultation.-Sept-2019.pdf). Coating of the islets with heparin has been shown to prevent instant blood-mediated inflammatory reaction following transplant in pigs [139]. However, culture of human islets in heparin has been shown to lead to an increased deposition of human islet amyloid polypeptide, which would impair β-cell function, despite also preventing β-cell apoptosis [137].

Applications of sulfation pathways research might result in enabling technologies. This may encompass preservation of (intracellular) islet levels of heparan sulfate during the islet isolation process to optimize islet survival after transplantation [36]. Coating the pancreatic islet surface with a chondroitin sulfate-branched-PEG nanocoating may improve transplant success [140], a strategy also applicable to other types of cell therapies. 3D-bioprinted double-crosslinked alginate/chondroitin sulfate patches might improve diabetic wound healing [141,142]. Finally, chondroitin sulfate-coated gold nanoparticles might enable oral delivery of insulin [143,144], with the potential of revolutionizing diabetes treatment.

## Conclusion

There is much evidence to suggest that sulfation pathways could be important in the maintenance of functional β-cell mass and regulation of β-cell function. This paper looks from the side of sulfation pathways on diabetes, discussing implications of heparan-sulfate and heparin, sulfatide and sulfo-cholesterol, various sulfated proteins, sulfo-steroid hormones and vitamin D-sulfate, as well as sulfated metabolites, on different types of diabetes and various aspects of β-cell physiology. A biotechnological section concludes this review, showing exciting new developments where sulfation pathways may be implemented in drug manufacturing and delivery, coated nanoparticles and advanced xeno-grafting and β-cell transplantation.

What else might be relevant for sulfation pathways in diabetes? More and more insulin variants become known, to put the evolution of insulin as a hormone into new perspectives, including viral insulin-like peptides. Certainly, it will be interesting to identify more and other sulfated proteins in β-cells and around them. Another route of research could involve tracing co-expression patterns of sulfation pathways genes in diabetes-related datasets. Finally, there are several molecules in sulfation pathways that await their biotechnical application, such as sulfatides for improving insulin injections.

Hopefully, a better understanding of the role of sulfation pathways in β-cells could lead to better understanding of the regulatory mechanisms involved, opening up new avenues to better treatment for diabetes.

## Summary

- More and more evidence suggest that sulfation pathways play a role in the maintenance of β-cell mass and regulation of β-cell function.
- Sulfation pathways including heparan-sulfate and heparin, sulfatide and sulfo-cholesterol, various sulfated proteins, sulfo-steroid hormones and vitamin D-sulfate, as well as sulfated metabolites, may be implicated in different types of diabetes.
- New developments in sulfation pathways research may be implemented in drug manufacturing and delivery, coated nanoparticles and advanced xeno-grafting and β-cell transplantation.
- A better understanding of the role of sulfation pathways in β-cells could lead to insights into the regulatory mechanisms involved, opening up new avenues to better treatments of diabetes.

## Abbreviations

FGF: fibroblast growth factor
IDS: iduronate 2-sulfatase
PAPS: 3′-phospho-adenosine-5′-phospho-sulfate
ROS: reactive oxygen species
